# Computer-based detection of depression and dementia in spontaneous speech

**DOI:** 10.1192/j.eurpsy.2021.936

**Published:** 2021-08-13

**Authors:** K. Chlasta, P. Holas, K. Wolk

**Affiliations:** 1 Department Of Information Technology, Polish-Japanese Academy of Information Technology, Warsaw, Poland; 2 Department Of Psychology, University of Warsaw, Warsaw, Poland

**Keywords:** machine learning, mental health monitoring, speech technology, prosodic analysis

## Abstract

**Introduction:**

There is a significant relation between old-age depression and subsequent dementia in patients aged 50. This supports the hypothesis of old-age depression being a predictor, and possibly a causal factor, of subsequent dementia. The number of people aged 60 years and over has tripled since 1950, reaching 16% in 2050, leading to new medical challenges. Depression is the most common mental disorder in older adults, affecting 7% of the older population. Dementia is the second most common with about 5% prevalence worldwide, but it is the first leading cause of disease burden.

**Objectives:**

Early detection and treatment is essential in promoting remission, preventing relapse, and reducing emotional burden. Speech is a well established early indicator of cognitive deficits. Speech processing methods offer great potential to fully automatically screen for prototypic indicators of both dementia and depressive disorders.

**Methods:**

We present two different methods to detect pathological speech with artificial neural networks. We use both deep architectures, as well as more traditional machine learning approaches.

**Results:**

The models developed using a two-stage deep architecture achieved 59% classification accuracy on the test set from DementiaBank. Our CNN system achieved the best classification accuracy of 63.6% for dementia, but reaching 70% for depressive disorders on the test set from Distress Analysis Interview Corpus.

**Conclusions:**

These methods offer a promising classification accuracy ranging from 63% to 70%, applicable in an innovative speech-based screening system.

